# Prion-Like Domains in Phagobiota

**DOI:** 10.3389/fmicb.2017.02239

**Published:** 2017-11-15

**Authors:** George Tetz, Victor Tetz

**Affiliations:** Human Microbiology Institute, New York, NY, United States

**Keywords:** prions, bacteriophage, bacterial cell, interaction, prion-like-domain, microbiota

## Abstract

Prions are molecules characterized by self-propagation, which can undergo a conformational switch leading to the creation of new prions. Prion proteins have originally been associated with the development of mammalian pathologies; however, recently they have been shown to contribute to the environmental adaptation in a variety of prokaryotic and eukaryotic organisms. Bacteriophages are widespread and represent the important regulators of microbiota homeostasis and have been shown to be diverse across various bacterial families. Here, we examined whether bacteriophages contain prion-like proteins and whether these prion-like protein domains are involved in the regulation of homeostasis. We used a computational algorithm, prion-like amino acid composition, to detect prion-like domains in 370,617 publicly available bacteriophage protein sequences, which resulted in the identification of 5040 putative prions. We analyzed a set of these prion-like proteins, and observed regularities in their distribution across different phage families, associated with their interactions with the bacterial host cells. We found that prion-like domains could be found across all phages of various groups of bacteria and archaea. The results obtained in this study indicate that bacteriophage prion-like proteins are predominantly involved in the interactions between bacteriophages and bacterial cell, such as those associated with the attachment and penetration of bacteriophage in the cell, and the release of the phage progeny. These data allow the identification of phage prion-like proteins as novel regulators of the interactions between bacteriophages and bacterial cells.

## Introduction

Prions were demonstrated to play important roles in eukaryotes and prokaryotes, and they have been intensively studied during the last decade (Alberti et al., 2009). Prion proteins (PrPs) are characterized by self-propagation, undergoing a conformational switch from one conformational state to another, which leads to the creation of new prions (Bolton et al., 1982; Telling et al., 1995). Pathologically, prions are characterized by a process in which the infectious form of prion (PrPSc) interacts with the endogenous PrPs, catalyzing the transformation of the endogenous molecule into misfolded PrPSc aggregates (Ma and Lindquist, 2002). This was first observed in mammals, and the pathological deposition of insoluble protein aggregates was shown to be associated with the development of a number of human diseases, including scrapie, Creutzfeldt-Jakob disease, Alzheimer’s disease, Parkinson’s disease, amyloidosis, and other (Collinge, 2001; Wemheuer et al., 2017).

One of the best-known forms of PrPs are amyloid proteins that, in addition to the role of infectious agents, were shown to be an important component of many physiological processes in eukaryotes and prokaryotes (Westergard et al., 2007; Sabate et al., 2015; Walker et al., 2016).

Although the amino acid sequences and function of these evolutionarily unrelated proteins are diverse, all amyloid fibrils can be misfolded into distinct conformers, forming highly ordered cross-β structures (Cheng et al., 2012).

Recently, these highly ordered parallel or antiparallel amyloid β-sheets were identified in yeasts and bacteria, suggesting that they may play an important physiological role (de Groot et al., 2012; Angarica et al., 2013; Malinovska et al., 2013). Unique mechanical and biological properties of amyloid sheets have multiple functions in a variety of bacterial species, and the secretion of amyloid proteins represents an important step during biofilm formation (Chapman et al., 2002; Fowler et al., 2007; Blanco et al., 2012). To discriminate between the physiological and pathological roles of amyloid, these forms that are involved in physiological process have been termed the functional amyloid (Chiti and Dobson, 2006). Therefore, the propagation of prions was shown to enable environmental adaptation, the activation of stress response, and in yeasts these molecules play important roles in the preservation of long-term memory (Shorter and Lindquist, 2005; Suzuki et al., 2012). The aggregation of PrPs is an amino-acid sequence-specific process, but the mechanism underlying the *de novo* appearance of prions remains unclear. PrPs are rich in asparagine (Q) and glutamine (N) domains, which was exploited for the development of an algorithm aimed at the identification of candidate prionogenic domains (PrDs) (Michelitsch and Weissman, 2000; Alberti et al., 2009). Currently, the most widespread PrD prediction algorithm is based on the hidden Markov model (HMM), the probabilistic sequence model based on the maximum likelihood estimation (Eddy, 1998; Angarica et al., 2013).

The HMM can be found at the core of several bioinformatic approaches to the statistical representation of prion domains, which allow the scoring of protein sequences according to the likelihood that these proteins are prions (Toombs et al., 2010; Lancaster et al., 2014). To that end, the log-likelihood ratio (LLR) score is used to evaluate the sets of amino-acid interactions, with a large LLR indicating a high similarity between the examined interaction sets, whereas the LLR is nearing zero when comparing the sets of random interactions. Using these web-based algorithms, prion-forming domains were extensively studied in mammalian, bacterial, and fungal proteomes, demonstrating that prions are common in different organisms, and represent important global regulators (Iglesias et al., 2015; Yuan and Hochschild, 2017).

In prokaryotes, PrPs were shown to play important roles in molecular transport, secretion, cell wall development, and other processes (Blanco et al., 2012; Yuan et al., 2014). Moreover, prion determinants were shown to participate in the inter-kingdom communication between prokaryotes and eukaryotes, leading to the alterations in the amyloidogenesis in *Caenorhabditis elegans* following its colonization with amyloid-producing bacteria (Chen et al., 2016).

However, the prion-like domains in viruses, in particular the bacterial viruses, bacteriophages, have not been thoroughly examined. Bacteriophages are ubiquitous organisms that are found almost everywhere, from the oceans, with the estimated 10^31^ viral particles, to mammalians, with over 10^15^ bacteriophages in the adult human gut (Dupuis et al., 2013; Dalmasso et al., 2014). The life cycle of bacteriophages can be lytic, resulting in bacterial death and *de novo* assembly of virions, or lysogenic, in which bacteriophage DNA is integrated into the bacterial genome. Both bacteriophage life cycles are important in the regulation of bacterial population. Phage-mediated horizontal gene transfer, occurring during the lysogenic cycle, is especially important for the bacterial ecological adaptation and is a common mechanism underlying the spreading of antibiotic resistance (Ochman et al., 2000). Moreover, we previously demonstrated that the bacteriophages, by altering mammalian gut flora, can induce an increase in gut permeability and facilitate endotoxemia, indirectly affecting the vital activities of microorganisms (Tetz and Tetz, 2016; Tetz et al., 2017). Previous studies revealed a correlation between the increased gut permeability and the development of a variety of human pathologies, including diabetes, neurodegenerative, and cardiovascular diseases. Therefore, the role of bacteriophages as the potential indirect causative agent inducing the development of these diseases requires further investigation (Bischoff et al., 2014).

Here, we studied in detail the putative prion domains in bacteriophages. We employed HHM algorithm for the analysis of bacteriophage proteomes, using the UniProtKB database. To the best of our knowledge, this is the most extensive effort aimed at the identification of candidate PrD sequences among bacteriophages. Furthermore, we analyzed the trends in the distribution of PrDs in different protein families and the correlations between bacteriophage families and the host bacterial host. The present predictive approach for the first time uncovers a large set of putative prionogenic proteins whose further experimental characterization might contribute significantly to understanding bacteriophages biology.

## Materials and Methods

### Protein Sequences

To identify the PrDs present in bacteriophage proteomes, protein sequences were obtained from the UniProt KnowledgeBase (Swiss-Prot and TrEMBL) (UniProt Consortium, 2012).

The protein functions were manually curated using the information from the UniProt database, the National Center for Biotechnology Information (NCBI) protein sequence database^1^, and the literature.

### Identification of PrDs in Bacteriophage Proteomes

The presence of PrDs was analyzed in all bacteriophage proteins using the prion-like amino acid composition (PLAAC) prion prediction program based on the HMM and trained using the known PrDs, by identifying the compositional bias toward N and Q residues. By employing a cutoff of 0.003 LLR, we identified 5040 PrDs (**Supplementary Table S1**). The enrichment values obtained for the proteins detected in the bacteriophage family subsets were compared with the total number of analyzed proteins.

We analyzed the regularities in the likelihood of the identified PrDs to be prions, their distribution among bacteriophage families, host bacteria, and protein functions. The obtained data were used to generate a heatmap with R-statistical computing ^2^, using the “levelplot” package. The color key indicates a range between the lowest (white) and the highest (red) LLR values.

### Statistical Analyses

All statistical analyses were performed using the statistics package Statistica for Windows (version 5.0). Data were compared between bacteriophage families by using a χ^2^ test or the Fisher’s exact test. Significant differences were calculated using one-way analysis of variance (ANOVA) with multiple comparisons and a standard confidence interval of 95%. A value of *p* < 0.001 was considered statistically significant in all tests. Correlations between the size of bacteriophage proteome and PrD enrichment were calculated using the Spearman’s rank correlation coefficient test.

## Results

### Identification of PrDs in Bacteriophage Proteomes

To identify the PrDs in bacteriophages using the PLAAC algorithm we analyzed 2111 bacteriophage proteomes comprising a total of 370,617 proteins retrieved from the UniProtKB database. We detected 5040 PrPs with the LLRs higher than 0.003 (1.35% of the total bacteriophage protein dataset) (Lancaster et al., 2014).

In **Table 1**, the trends in the PrD distribution across different bacteriophage families are presented. PrD frequency ranged from 1.16 to 4.49% when analyzing bacteriophage families with at least 400 sequenced proteins. Due to the low representation of archaeal phages, it was not possible to compare them with the bacteriophage proteins with sufficient reliability. Therefore, we focused on the analysis of the PrDs in bacteriophages. Within Caudovirales, the most well-studied order of phages, the highest numbers of proteins containing the PrDs when analyzing bacteriophage families with more than 10,000 sequenced proteins were found among *Podoviridae*, with over 1.74% of the total *Podoviridae* proteins were shown to harbor PrDs (*p* < 0.001), constituting 4.21% of the total detected PrDs among Caudovirales (**Table 1** and **Supplementary Table S1**).

**Table 1:** Summary of the prion predicted in different bacteriophage families.

Phage order	Phage family	Host	# Proteins	# PrD predictions	% of the proteome
Undefined	*Ampullaviridae*	Archaeal	173	6	3,46
Undefined	*Bicaudaviridae*	Archaeal	445	20	4,49
Undefined	*Fuselloviridae*	Archaeal	404	10	2,47
Undefined	*Globuloviridae*	Archaeal	87	1	1,14
*Ligamenvirales*	*Rudiviridae*	Archaeal	941	18	1,91
Undefined	*Lipothrixviridae*	Bacterial	599	7	1,16
Undefined	*Inoviridae*	Bacterial	1073	46	4,28
Undefined	*Leviviridae*	Bacterial	1082	52	4,8
Undefined	*Corticoviridae*	Bacterial	21	1	7,76
Undefined	*Microviridae*	Bacterial	2827	35	1,23
*Caudovirales*	*Myoviridae*	Bacterial	166120	2425	1,45
*Caudovirales*	*Podoviridae*	Bacterial	38922	678	1,74
*Caudovirales*	*Siphoviridae*	Bacterial	157433	1665	1,05
Undefined	*Tectiviridae*	Archaeal/Bacterial	490	20	4,08
Undefined	Undefined	Bacterial	N/A	19	N/A

The analysis shown in **Supplementary Table S1** demonstrates an interesting trend of representation of prionogenic domains among different bacteriophages families and host organisms.

**FIGURE 1:**
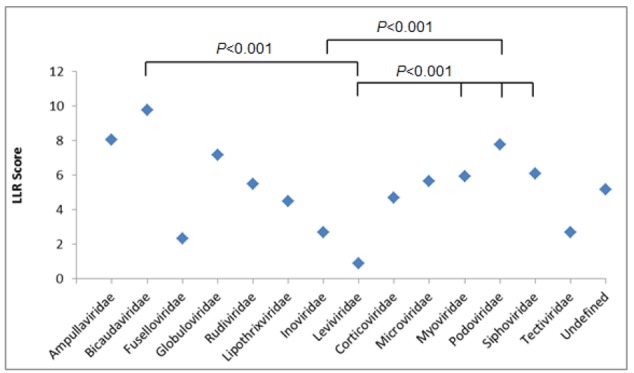
Log-likelihood ratio (LLR) score showing the predicted putative PrDs across bacteriophage families. Results were analyzed using one-way ANOVA.

Next, we determined the patterns of correlation between bacteriophage families and LLR scores (**Figure 1** and **Supplementary Table S2**).

The average numbers of LLRs between three most abundant bacteriophage families, *Myoviridae, Podoviridae*, and *Siphoviridae*, were similar, with the highest LLR scores obtained for *Podoviridae* family, with an average LLR of 7.78 (*p* < 0.001) (**Supplementary Tables S3A–C**).

Under the same conditions, we compared the LLRs between the remaining bacteriophage families, and *Myoviridae* were shown to have the highest number of PrDs, with the LLR score over 30. We identified 114 PrDs with the medium LLR score over 20 within *Myoviridae* family, yielding 3.29% of the total number of PrDs identified in this bacteriophage family (Supplementary Figure S1).

The same trend was observed when analyzing top 100 scoring PrDs. Over 60% of these 100 PrDs were derived from *Myoviridae* bacteriophages, with the medium LLR score of 40.55 (**Supplementary Table S4**).

Afterward, we aimed to select bacteriophage proteins in bacteria that may have a greater prion-forming potential. To this end, we analyzed the correlation between LLR score of the PrD proteins and the host bacteria. The average LLR score varied significantly between the bacteriophages of different bacterial species. The lowest mean LLR values were detected among *Cellulophaga* and *Escherichia* bacteriophages (3.49 and 3.98, respectively) and the highest LLR score was obtained for the *Bacillus* bacteriophages, with the average LLR of 8.96 (*p* < 0.001).

**FIGURE 2:**
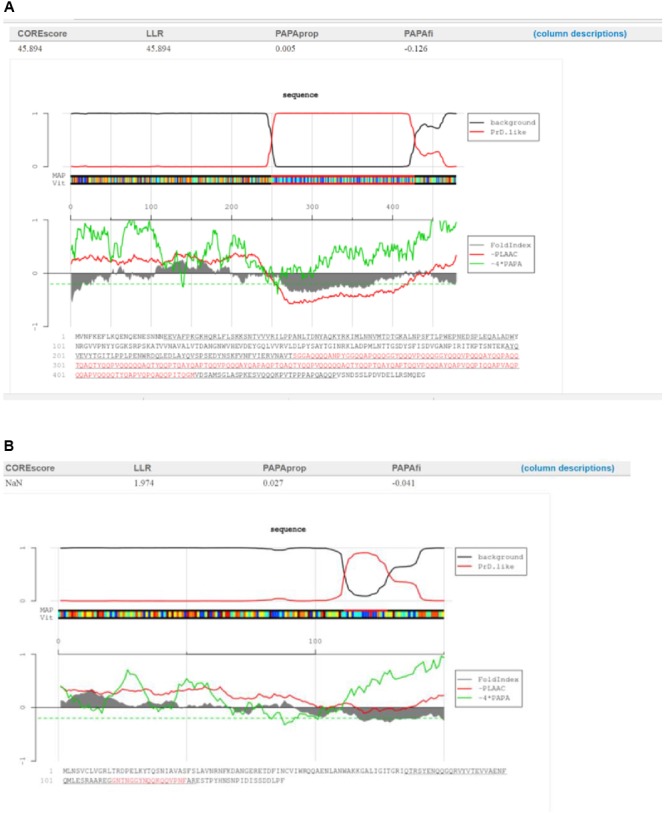
Characterization of the candidate bacteriophage PrDs in the single-stranded DNA binding proteins. **(A)**Graphical representation of the highest LLR value in this group of proteins identified in the *Listeria* phage LMSP-25. **(B)** Graphical representation of the lowest LLR value in this group of proteins in the *Streptococcus* phage 5093. The predicted PrDs are highlighted in red.

No direct correlation was observed between the functions of proteins containing PrDs and the LLR score. One of both highest and lowest LLR scores were obtained for the single-stranded DNA binding protein of *Listeria* bacteriophage LMSP-25 (LLR score, 45.89; *Myoviridae*) and the *Streptococcus* bacteriophage 5093 (LLR 1.97; *Siphoviridae*), respectively (**Figures 2A,B**).

Additionally, we analyzed the proteome enrichment with PrDs in the bacteriophages of different bacteria. The highest enrichment rate, with at least five PrDs per proteome, was found among the bacteriophages of *Bacillus, Cronobacter, Lactobacillus, Synechococcus, Staphylococcus*, and other bacteria (**Figure 4** and **Supplementary Table S5**). The majority of these bacteriophages containing more than five PrDs per one proteome was shown to belong to the *Myoviridae* family. Since the members of this family have larger proteomes compared with those of other bacteriophage families, we analyzed and obtained a direct correlation between the number of PrDs per bacteriophage and the proteome (*p* < 0.05), but no correlations were observed within any bacteriophage family (**Supplementary Table S6**). Notably, the particular enrichment with PrDs was found across the majority of *Prochlorococcus* and *Synechococcus* cyanophages identified as PrD carriers in this study.

### Structural Domains Associated with to Bacteriophage PrDs

**FIGURE 3:**
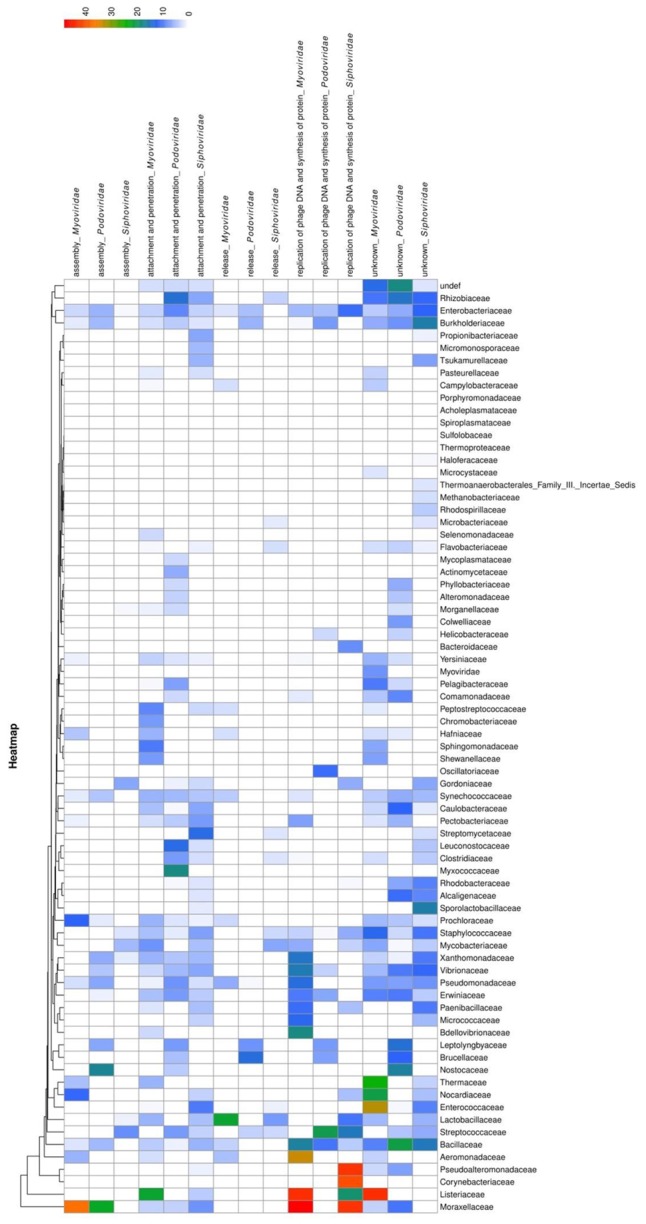
Heatmap showing PrD distribution in bacteriophages. The correlation between LLR score of the identified PrDs, their distribution across bacteriophage families, host bacteria, and their function in the bacteriophage-bacterial interaction are presented. The likelihood that an identified PrD is a prion is represented by a color scale, from white to red (maximum).

To analyze the functions of bacteriophage proteins with PrDs, we clustered the identified domains into five groups, according to the major steps during the process of bacteriophage interactions with its host cell (Marvin, 1998). The proteins were grouped based on their functions during the interactions with the bacterial host cells into the following groups: attachment and penetration, replication of bacteriophage DNA and protein synthesis, assembly, release, and unknown functions (Laemmli, 1970; Zylicz et al., 1989; Marvin, 1998; Rakhuba et al., 2010; Aksyuk and Rossmann, 2011). The obtained correlations between the LLR scores, bacterial and bacteriophage families, and protein functions were analyzed and are displayed as a heatmap in **Figure 3**.

The most abundant (2046 PrPs; medium LLR score 4.86) functional group was shown to be the attachment and penetration group, which included the proteins associated with the host cell binding and genome injection. We identified receptor binding proteins, tail fiber proteins, peptidase_M23 baseplate wedge components, lysins, and other proteins known to have crucial functions in the various aspects of phage infection, including adsorption, peptidoglycan hydrolysis, cell wall penetration, and DNA ejection, but the PrDs were predominantly found among tail component, tail fiber, and baseplate proteins (Rodríguez-Rubio et al., 2012; Stockdale et al., 2013). Furthermore, we identified a PrD in gp98/99 proteins that form the cell-puncturing device of *Mycobacterium* phages. Some of the identified proteins associated with surface structures, such as tail tube cap, are not involved in the direct contact with the bacterial cell, but they represent important structural components allowing the injection of DNA (Arisaka et al., 2003; Orlova, 2012). Heatmap analysis results showed that *Podoviridae* belonging to the *Mycobacteriaceae* family and *Myoviridae* of *Listeriaceae* have the highest LLR score (**Figure 3**).

The second largest group was shown to contain 390 PrDs (medium LLR score, 7.93) involved in the replication of phage DNA and protein synthesis. We identified several *Erf* proteins, essential for bacteriophage DNA recombination, proteins belonging to glycosyltransferase family protecting bacteriophage DNA from degradation, and single-strand binding proteins (Markine-Goriaynoff et al., 2004). Other identified proteins included those involved in the regulation of host cell metabolism, such as serine/threonine phosphatases (Howard-Varona et al., 2017). The highest average LLR score was obtained for the members of *Myoviridae* family of *Moraxellaceae* and *Listeriaceae* (**Figure 3**).

Following this, we analyzed proteins with PrDs that are associated with the bacteriophage assembly in the host cells (170 proteins; medium LLR score, 6.16). These proteins were identified in all major families of Caudovirales. The highest average LLR score was obtained for the proteins of *Myoviridae* family of *Moraxellaceae* (**Figure 3**). These proteins are also associated with the organization and maturation of bacteriophages, and those involved in the tail assembly and DNA packaging (Linderoth et al., 1994; Boudko et al., 2002; Aksyuk and Rossmann, 2011).

The identified PrDs in proteins that are involved in cell wall lysis and the release of bacteriophages from the host cells were less abundant and this group contained only 105 proteins (medium LLR score, 5.13). This group predominantly comprised lysins, including those with the amidase activity and proteins with peptidoglycan binding functions (hydrolases, D-ala-D-ala carboxypeptidase, endopeptidase CHAP domain protein, and others), which are involved in bacterial cell wall degradation, resulting in cell lysis and release of progeny viruses. They were predominantly detected among *Siphoviridae* infecting *Streptococcaceae* and *Mycobacteriaceae*. The highest average LLR score was obtained in *Myoviridae* family of *Moraxellaceae* (**Figure 3**).

Finally, we identified 2329 PrDs among the proteins with the still unknown functions. Among these proteins in *Staphylococcus* bacteriophages belonging to the *Siphoviridae* family, we identified Panton-Valentine leukocin (LukS-PV), a cytotoxin associated with the increased virulence of *Staphylococcus aureus*, which can induce a considerable tissue damage (Vandenesch et al., 2003). This is in agreement with previous observations that PrDs in viruses play important roles in the determination of virulence potential (Iglesias et al., 2015).

## Discussion

To the best of our knowledge, this is the first analysis of PrDs among bacteriophages. The results of our study underline the necessity of characterizing bacteriophages and we propose here that the all bacteriophage genes should be known as the phagobiome.

Here, PrDs were more frequently found among archaeal phages, and the analysis of the three best-known Caudovirales families showed that these domains are more abundant among *Podoviridae* than *Myoviridae* or *Siphoviridae*.

Furthermore, our analyses demonstrated that approximately 50% of all sequenced bacteriophages available in public databases contain at least one PrD. The majority of bacteriophages contain less than five PrDs per proteome. Surprisingly, the vast majority of bacteriophages with more than five PrDs per proteome were shown to belong to the *Myoviridae* family. Since the total numbers of PrDs found in *Myoviridae* (2425) and *Siphoviridae* (1665) are of the same order of magnitude, these further supports the enrichment of PrDs in the *Myoviridae* proteomes. However, *Myoviridae* members have larger proteomes than the members of *Siphoviridae* family, which may explain the observed differences in the PrD enrichment (Sandaa, 2009). Further analyses indicated a direct correlation between the proteome size and the PrD enrichment, and therefore, we further explored whether the proteome size of bacteriophages in the *Myoviridae* family is related to the PrD enrichment. However, we have not observed a linear correlation between these parameters, suggesting that the PrD enrichment does not depend only on the size of a *Myoviridae* proteome.

Moreover, the majority of the top 100 scoring PrDs was also detected in the members of *Myoviridae* family, and among these, several short protein sequences were found to harbor a higher number of Q-rich domains than the longer ones. This indicates that the scores obtained in the analysis of protein sequences according to their prion-like characteristics do not correlate only with the size of a protein. However, the functional relevance of these findings remains unclear, since the majority of these proteins have not been characterized. The analysis of proteins with the known functions showed that the proteins involved in the replication of bacteriophage DNA and protein synthesis have the largest LLR scores.

Many PrD-containing proteins with known functions were identified among *Bacillus* phages that infect the spore-forming *Bacillus* spp. The identified PrDs were found in proteins responsible for attachment and penetration, replication of bacteriophage DNA, and release within the same *Bacillus* phages. Furthermore, the LLR scores of PrDs in the *Bacillus* phages were shown to have the highest meaning of the prion likelihood ratio compared with the phages associated with other bacterial genera. Previously, we reported a particular role of spore-forming bacteria in the global microbiota, which allowed their identification as the members of sporobiota (Tetz and Tetz, 2017). Taken together, previous results and the ones obtained in this study may suggest that the global distribution of sporobiota members and *Bacillus* spp., and their increased transfer between the ecological niches is associated with the presence of resistant transmissible spores due to their interactions with bacteriophages (von Wintersdorff et al., 2016).

**FIGURE 4:**
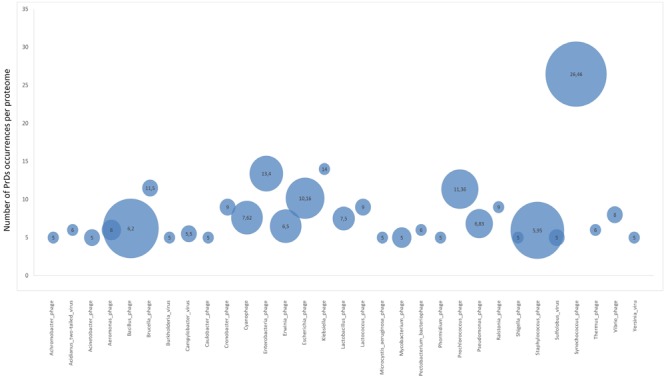
Proteome enrichment for PrDs across bacteriophages. Bubble charts represent the medium enrichment rate with PrDs per bacteriophage proteome (phages with at least five PrDs per proteome have been considered) and the number of phages in which these PrDs were identified. The values in the bubbles are the median number of PrDs occurring per proteome in each bacteriophage group. Bigger bubble size indicates higher number of phages with at least five PrDs per proteome, and smaller size indicates lower number of phages with at least five PrDs per proteome.

However, the highest numbers of PrDs per proteome were found across *Prochlorococcus* and *Synechococcus* phages, whose hosts are represented by evolutionary ancient Cyanobacteria (**Figure 4**) (Schirrmeister et al., 2011). This finding along with the higher prevalence of PrDs in archaea suggests the possible association of the presence of PrDs and “evolutionary age,” however, this requires an independent analysis. Regardless, the enrichment of PrDs in the most abundant phages on Earth infecting marine *Prochlorococcus* and *Synechococcus* suggests the possible particular role of these elements (Sullivan et al., 2010).

To determine the potential correlations between the functions of PrD-containing proteins, bacteriophage families, and host organisms, we generated a heatmap that showed that the highest LLRs were found among *Myoviridae* and *Siphoviridae* members of *Moraxellaceae, Listeriaceae*, and *Pseudoalteromonadaceae*, which were shown to be associated with replication of phage DNA, protein synthesis, and bacteriophage assembly. However, these data do not correspond to the frequency of PrD in bacteriophage proteomes. A vast majority of the bacteriophage PrD-containing proteins are involved in the interactions between a bacteriophage and bacterial cell wall, their attachment and penetration, and the release of progeny phages.

Attachment and penetration represent crucial steps in the bacteriophage–bacterium interaction, since these processes allow the entry of the bacteriophage DNA inside the host. We identified PrDs in the proteins forming different structural components of bacteriophages, including head, neck, sheath, baseplate and fibers, for tailed viruses, receptor-binding proteins for tailless *Tectiviridae*, and in the coat proteins III and IV of the filamentous *Inoviridae* (Hulo et al., 2017). This indicates the role of the identified PrDs in the formation of surface structures and specific and non-specific attachments to bacterial cells (Spinelli et al., 2014). Moreover, a similar trend in the distribution of prion-like domains in proteins responsible for interactions and virulence was defined by Gene Ontology GO terms across bacteria (Iglesias et al., 2015). Notably, PrDs were common across different bacterial and bacteriophage families, indicating the universal regulatory role of these putative prion domains (Iglesias et al., 2015).

Furthermore, since we identified PrDs in the interacting proteins in different bacteriophage families infecting a variety of bacterial hosts, this implies that these PrDs are conserved across species. However, since the PrDs in the surface proteins were not identified in all analyzed bacteriophages, and in those where they were identified, PrDs were found in some, but not all surface proteins of the given phage, this suggests that the presence of PrD-containing proteins may be beneficial, but it is not obligatory. The attachment of bacteriophages is a complex process, accompanied by a number of bacteriophage-resistance responses initiated in a bacterial cell (Labrie et al., 2010). Our findings may indicate that the proteins with the PrDs can alter their conformational states, adding further complexity and specificity in the phage-bacterial interactions, and this may serve as a mechanism to overcome some bacterial resistance mechanisms. Moreover, it can be speculated that the PrDs in the interacting proteins may be involved not only in the interactions with host bacteria, but also in the interactions of bacteriophages with other components of microbial communities. These observations agree with our recent studies showing that the oral administration of bacteriophages induces an increase in the intestinal permeability and endotoxemia in mammals, significantly changing microbiota composition, beyond the possible direct effect of bacteriophages, and because of this, it was suggested that these effects are exerted through yet unknown mechanisms (Tetz and Tetz, 2016; Tetz et al., 2017).

Additionally, the results obtained in this study suggest that the presence of PrDs in bacteriophages may be associated with a number of mammalian diseases, in which the role of prions was demonstrated both in macroorganisms and in gut bacteria. Thus in the article by Chen et al. (2016), *C. elegans* fed with the prion-producing *Escherichia coli* was shown to have an enhanced prion aggregation in brain. It can be assumed that a similar process may be observed following the introduction of bacteriophages and that bacteriophages contribute to the observed processes.

Proteins that are involved in the bacterial cell wall interactions and the release of progeny phages from the host were shown to harbor PrDs as well. The release stage involves bacterial cell wall degradation from within the cells with bacteriophage-encoded peptidoglycan hydrolases, synthesized at the end of the multiplication cycle. We identified PrDs in phage-encoded endolysins, and the majority of these proteins was found in *Siphoviridae* family, with only a few detected among the members of *Podoviridae* and *Myoviridae*.

Some of the proteins enriched in the PrDs are involved in the replication of bacteriophage DNA and protein synthesis. In eukaryotes, PrD-containing proteins were shown to play a role in the interactions and binding with the nucleic acids (King et al., 2012). Nucleic acids were shown to play a crucial role in the PrP conversion into PrPSc-like species as cofactors (Macedo and Cordeiro, 2017).

In bacteriophages, we determined that the presence of PrDs can also be associated with the single-strand binding proteins, known as essential components of bacteriophage DNA synthesis. Therefore, this indicates that these PrDs can be easily misfolded into PrPSc through the interactions with the DNA, which was shown to be an important component inducing this misfolding (Cordeiro et al., 2001). This type of interaction with the host cell may represent an additional process through which the bacteriophages control bacterial intracellular functions (Wagner and Waldor, 2002; Davies and Davies, 2010).

Bacteriophages are important vehicles facilitating the genetic exchange between microorganisms, including the spreading of virulence factors and the antibiotic resistant genes (Lina et al., 1999). Here, we identified a PrD in the LukS-PV protein of some Siphoviridae members and unclassified phages. This cytotoxin can be found among methicillin-resistant *S. aureus* and belongs to the group of β-pore-forming toxins (Vandenesch et al., 2003). The presence of LukS-PV is associated with the increased virulence of *S. aureus*, leading to abscess formation and severe necrotizing pneumonia. The detected PrDs located in LukS-PV indicates the role of these domains in the virulence potential of the cytotoxins and their role in the pathogenicity. Our results may indicate that the presence of PrDs in LukS-PV affects its interactions with the membranes of the targeted eukaryotic cells and induce the pore-forming potential, which is supported by previous studies (Iglesias et al., 2015).

Taken together, we identified numerous putative PrD-containing proteins in bacteriophages. We observed consistent patterns of the distribution of PrDs across different bacteriophage families. However, since the infectious agents of some bacteria were shown to lack PrDs, this may indicate that the diversity of phagobiota and phogobiome is underestimated. Although bacteriophage genomes are significantly smaller than bacterial genomes, currently less than 2,500 phage genomes are deposited in the NCBI database, compared with almost 90,000 bacterial whole genome sequences.

Although several trends in the frequency of PrDs across bacteriophage families have been detected, future studies are required (Pourcel et al., 2017), to elucidate further the functions and presence of PrP-containing proteins in bacteriophages. The predictive approach employed here revealed for the first time a large set of putative PrPs, and further experimental characterization of these proteins may contribute to the understanding of bacteriophage biology.

## Author Contributions

GT and VT designed and conducted the experiments and analyzed data, GT wrote the manuscript.

## Conflict of Interest Statement

The authors declare that the research was conducted in the absence of any commercial or financial relationships that could be construed as a potential conflict of interest.

## Footnotes

1 http://www.ncbi.nlm.nih.gov/

2 www.r-project.org

## Supplementary Material

TABLE S1 LLR score showing the predicted putative PrDs across bacteriophages.

TABLE S2 Summary of the LLR score of prion predictions across different phage families.

TABLE S3 Least squares means for effect of bacteriophage family on LLR score.

TABLE S4 Top 100 scoring PrDs across bacteriophages.

TABLE S5 Proteome enrichment for PrDs across bacteriophages.

TABLE S6 Correlation between the number of PrD per phage and the proteome size.
