# Supplementary material for: Prion-Like Domains in Phagobiota
Source: Front Microbiol. 2017 Nov 15;8:2239. doi: 10.3389/fmicb.2017.02239 (PMC5694896; doi:10.3389/fmicb.2017.02239)
Supplement: TABLE S2 — Summary of the LLR score of prion predictions across different phage families. [file Table_2.pdf]

Supplementary Table 2

Summary of the LLR score of prion predictions across different phage families

Phage Order	Phage Family	Host	LLR Score
Undefined	*Ampullaviridae*	Archeal	8.033500
Undefined	*Bicaudaviridae*	Archeal	9.782100
Undefined	*Fuselloviridae*	Archeal	2.333500
Undefined	*Globuloviridae*	Archeal	7.176000
Ligamenvirales	*Rudiviridae*	Archeal	5.505778
Undefined	*Lipothrixviridae*	Bacterial	4.488143
Undefined	*Inoviridae*	Bacterial	2.690261
Undefined	*Leviviridae*	Bacterial	0.863692
Undefined	*Corticoviridae*	Bacterial	4.680000
Undefined	*Microviridae*	Bacterial	5.643914
Caudovirales	*Myoviridae*	Bacterial	5.908475
Caudovirales	*Podoviridae*	Bacterial	7.780928
Caudovirales	*Siphoviridae*	Bacterial	6.080153
Undefined	*Tectiviridae*	Archeal/ Bacterial	2.699050
Undefined	Undefined	Bacterial	5.177220
