# Supplementary material for: Prion-Like Domains in Phagobiota
Source: Front Microbiol. 2017 Nov 15;8:2239. doi: 10.3389/fmicb.2017.02239 (PMC5694896; doi:10.3389/fmicb.2017.02239)
Supplement: TABLE S3 — Least squares means for effect of bacteriophage family on LLR score. [file Table_3.pdf]

Supplementary Table 3a

Source	DF	Type III SS	Mean Square	F Value	Pr > F
Family	2	1920.410170	960.205085	16.84	<.0001

Supplementary Table 3b
95% Confidence interval of bacteriophage families

	Family	LLR Score LSMEAN	95% Confidence Limits	
1	*Myoviridae*	5.908475	5.608805	6.208146
2	*Podoviridae*	7.780928	7.212436	8.349420
3	*Siphoviridae*	6.080153	5.717382	6.442923

Supplementary Table 3c
Least squares means for effect of bacteriophage family on LLR score

Least Squares Means for effect Family
Pr > |t| for H0: LSMean(i)=LSMean(j)
Dependent Variable: LLR Score

i/j	*Myoviridae*	*Podoviridae*	*Siphoviridae*
1		<.0001	0.7545
2	<.0001		<.0001
3	0.7545	<.0001	
