# Supplementary material for: Prion-Like Domains in Phagobiota
Source: Front Microbiol. 2017 Nov 15;8:2239. doi: 10.3389/fmicb.2017.02239 (PMC5694896; doi:10.3389/fmicb.2017.02239)
Supplement: TABLE S6 — Correlation between the number of PrD per phage and the proteome size. [file Table_6.pdf]

Supplementary Table 6

Correlation between the number of PrD per phage and the proteome size

All famalies	Correlation Coefficient Spearman's rho	.159
	Sig. (2-tailed)	.040
	N	168
Bicaudaviridae	Correlation Coefficient Spearman's rho	-.866
	Sig. (2-tailed)	.333
	N	3
Inoviridae	Correlation Coefficient Spearman's rho	
	Sig. (2-tailed)	.
	N	2
Leviviridae	Correlation Coefficient Spearman's rho	
	Sig. (2-tailed)	.
	N	2
Myoviridae	Correlation Coefficient Spearman's rho	.067
	Sig. (2-tailed)	.433
	N	137
Podoviridae	Correlation Coefficient Spearman's rho	.050
	Sig. (2-tailed)	.870
	N	13
Siphoviridae	Correlation Coefficient Spearman's rho	-.183
	Sig. (2-tailed)	.638
	N	9
Tectiviridae	Correlation Coefficient Spearman's rho	
	Sig. (2-tailed)	
	N	1
Undef	Correlation Coefficient Spearman's rho	
