# Supplementary material for: Prion-Like Domains in Phagobiota
Source: Front Microbiol. 2017 Nov 15;8:2239. doi: 10.3389/fmicb.2017.02239 (PMC5694896; doi:10.3389/fmicb.2017.02239)
Supplement: Supplementary file 7 [file Image_1.PDF]

Supplementary Figure 1

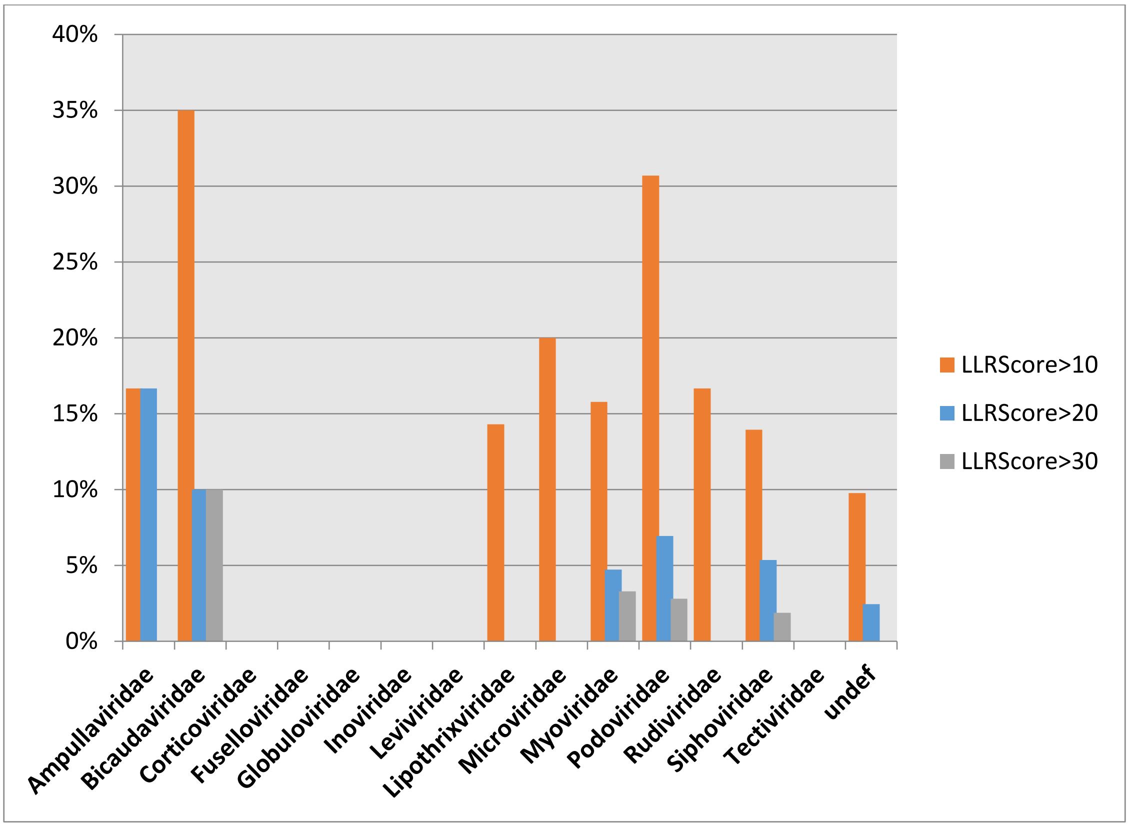

Supplementary Figure 1. Distribution of bacteriophage families with the LLR scores higher than 10, 20, and 30.
